# Intradermal Immunization with Wall Teichoic Acid (WTA) Elicits and Augments an Anti-WTA IgG Response that Protects Mice from Methicillin-Resistant *Staphylococcus aureus* Infection Independent of Mannose-Binding Lectin Status

**DOI:** 10.1371/journal.pone.0069739

**Published:** 2013-08-02

**Authors:** Kazue Takahashi, Kenji Kurokawa, Patience Moyo, Dong-Jun Jung, Jang-Hyun An, Lorencia Chigweshe, Elahna Paul, Bok Luel Lee

**Affiliations:** 1 Department of Pediatrics, Massachusetts General Hospital, Harvard Medical School, Boston, Massachusetts , United States of America; 2 Division of Pediatric Nephrology, Massachusetts General Hospital, Harvard Medical School, Boston, Massachusetts , United States of America; 3 National Research Laboratory of Defense Proteins, College of Pharmacy, Pusan National University, Busan, Korea; National Institutes of Health, United States of America

## Abstract

The objectives of this study were to investigate the immune response to intradermal immunization with wall teichoic acid (WTA) and the effect of MBL deficiency in a murine model of infection with methicillin-resistant *Staphylococcus aureus* (MRSA). WTA is a bacterial cell wall component that is implicated in invasive infection. We tested susceptibility to MRSA infection in wild type (WT) and MBL deficient mice using two strains of MRSA: MW2, a community-associated MRSA (CA-MRSA); and COL, a healthcare-associated MRSA (HA-MRSA). We also performed *in vitro* assays to investigate the effects of anti-WTA IgG containing murine serum on complement activation and bacterial growth in whole blood. We found that MBL knockout (KO) mice are relatively resistant to a specific MRSA strain, MW2 CA-MRSA, compared to WT mice, while both strains of mice had similar susceptibility to a different strain, COL HA-MRSA. Intradermal immunization with WTA elicited and augmented an anti-WTA IgG response in both WT and MBL KO mice. WTA immunization significantly reduced susceptibility to both MW2 CA-MRSA and COL HA-MRSA, independent of the presence of MBL. The protective mechanisms of anti-WTA IgG are mediated at least in part by complement activation and clearance of bacteria from blood. The significance of these findings is that 1) Intradermal immunization with WTA induces production of anti-WTA IgG; and 2) This anti-WTA IgG response protects from infection with both MW2 CA-MRSA and COL HA-MRSA even in the absence of MBL, the deficiency of which is common in humans.

## Introduction

*Staphylococcus aureus*, a Gram-positive bacterium, is a common commensal organism on skin and mucosa that can also cause serious infections in the skin and other soft tissues, bones and blood [1]. The morbidity and mortality of *S. aureus* infection have greatly increased with the rapid emergence of a much more virulent and antibiotic resistant strain that is identified by its resistance to the antibiotic methicillin, and which is therefore known as methicillin-resistant *S. aureus* (MRSA) [1], [2], [3]. All strains of *S. aureus*, including MRSA, are covered by a thick cell wall containing peptidoglycan, lipoteichoic acid (LTA) and wall teichoic acid (WTA) [4], [5]. WTA of *S. aureus* is a glycopolymer that covalently links to peptidoglycan. It is composed of an *N*-acetylmannosamine (ManNAc)-(β-1,3)-*N*-acetylglucosamine (GlcNAc) disaccharide with two glycerol phosphates, followed by 10 to 40 ribitol phosphate repeating units [4], [6]. WTA has been identified as an adhesion molecule that binds to host cells and promotes bacterial colonization and tissue invasion [7], [8], [9], [10].

The host defense system has two principal divisions, innate immunity and adaptive immunity, which are tightly coordinated in their functions. The innate immune system consists of innate immune cells, such as epithelial cells and phagocytes, and soluble factors such as complement proteins, coagulation factors and pattern recognition molecules, including mannose-binding lectin (MBL) [11]. Although genetic deficiency of MBL in mice increases infection susceptibility to certain pathogens, including *S. aureus*, this association is less apparent in humans, particularly in adults [12], [13], [14], [15].

Differences in antigen presentation, including the route of exposure, the context of antigen preparation, and adjuvant factors, are important determinants of host immune responses. It has been found in prior investigations that immunization of various vaccines by an intradermal route can elicit an effective immune response that protects from infection, whereas subcutaneous immunization does not elicit a protective immune response [16], [17], [18], [19]. Another relevant finding from our previous work is that commercially available WTA can be contaminated with other pathogen components, including LTA and peptidoglycan, which are known to modulate other pathways [20], [21].

We have recently discovered that healthy humans (that is, with no known pathogenic infection) have WTA-specific IgG in their blood, and that in purified form this anti-WTA IgG enhances the opsonophagocytic killing of *S. aureus* [21], [22]. Moreover, anti-WTA IgG competes with MBL for *S*. *aureus* binding, and it activates the classical complement pathway [21]. Anti-WTA IgG may also be protective since WTA has been found to induce abscess formation when it is subcutaneously injected with a foreign body [10], [23]. Consequently, these findings support the hypothesis that stimulating and enhancing an anti-WTA IgG response would help to eliminate bacteria and to reduce abscess formation.

In this study, we investigated whether intradermal WTA immunization would induce the anti-WTA IgG response, and whether this response was protective from infection with two strains of MRSA, COL HA-MRSA and MW2 CA-MRSA. In addition, considering the interaction between WTA and MBL, and given the high prevalence of MBL deficiency in humans, we examined whether the presence of MBL altered the anti-WTA IgG response and the efficacy of anti-WTA immunity in MRSA infection, using both *in vivo* and *in vitro* systems.

## Materials and Methods

### Purification of *S. aureus* WTA

WTA was prepared using previously reported methods [21], [22]. Briefly, WTA was purified from *S. aureus* strain T384, which is deficient in both a peptidoglycan *O*-acetyltransferase (*oatA*) gene and a lipoprotein diacylglycerol transferase (*lgt*) gene from *S. aureus* strain RN4220 [24]. The *lgt* mutation results in the absence of bacterial lipoproteins [20], and so WTA purified from the *S. aureus* strain T384 is not contaminated with bacterial lipoproteins. The *oatA* mutation makes *S. aureus* peptidoglycan sensitive to lysozyme. WTA attached to insoluble peptidoglycan was cleaved by treatments with lysostaphin and lysozyme. The solubilized WTA linked to a monomeric unit of peptidoglycan was purified with HiTrap-Q column chromatography. Complete digestion of polymeric peptidoglycan was confirmed by loss of melanization activity in insect hemolymph [25], demonstrating that the purified WTA did not contain polymeric peptidoglycan. Purified WTA was dissolved in PBS and aliquots were stored at −80° C.

### WTA immunization

MBL KO mice were generated and backcrossed on to a C57B/6J genetic background as described previously [12], [26]. All mice used in this study were 6–8 weeks old and were maintained in a specific pathogen free (SPF) environment. All animal experiments were performed under a protocol approved by the Subcommittee on Research Animal Care at the Massachusetts General Hospital.

Immunization experiments were performed using previously described methods with minor modifications [27]. Briefly, the purified WTA (5 µg in 50 µl PBS) was injected intradermally into the ventral skin using a 30 G needle (BD Biosciences) attached to a 1 ml syringe (BD Biosciences). The dose was calculated based on results from our previous studies [27], [28]. A control group was injected with 50 µl PBS in a similar manner. WTA or PBS control was injected on days 0, 20, 40, and 60. 10 days after each immunization, serum was collected by making a shallow cut in the tail vein and stored at −80° C. Day 0 serum collections were performed on day -5. Anti-WTA IgG titers were determined using a previously described ELISA method with minor modifications [27]. Briefly, a 384 well plate was coated with 2 µg of purified WTA and incubated with serially diluted mouse serum. Bound mouse IgG was detected using alkaline phosphatase-conjugated anti-mouse IgG (Promega) with *p*-nitrophenylphosphate substrate (pNPP, Sigma-Aldrich). A standard curve was generated from using murine IgG of known concentration. The chromogenic reaction was assayed by measuring absorbance (OD) at 405 nm (Molecular Devices).

### Infection studies *in vivo*

We chose a murine model of abscess formation following systemic bacterial infection because *S. aureus* is known to cause abscesses in a process mediated at least in part by WTA [10], [23]. In the model, abscesses (Figure S1) developed following systemic inoculation of sub-lethal inoculums of bacteria (bacteremia). Two *S. aureus* strains, COL HA-MRSA and MW2 CA-MRSA, were obtained from Dr. David C. Hopper of the Infection Control Unit at Massachusetts General Hospital and prepared as previously described [12]. Briefly, bacteria were grown in Columbia broth supplemented with 2% NaCl and harvested in the mid log phase. Washed bacteria were resuspended at 5×10^6^ colony forming units (cfu)/ml in saline and 0.2 ml of the suspension was intravenously injected. On day 10, mice were euthanized and the lung, liver, spleen, heart, and kidneys were examined for abscess formation. Optimal inoculation dosing and the time course for abscess formation were determined in our preliminary studies. Two kidneys from each mouse were combined and homogenized with 1 ml of PBS. Serially diluted homogenates were cultured in duplicate on tryptic soy agar (TSA) plates and resulting *S. aureus* colonies were counted.

### Complement activation assays

Complement activation was assayed by C4c deposition activity using previously described methods with minor modifications [12], [29]. C4c is cleaved from C4 upon activation of the classical pathway. Briefly, a 384 well assay plate was coated with purified WTA (1 µg/well) and blocked. Mouse serum diluted 1∶100,000 in tris buffered saline was added to each well and incubated in 20 µl total volume. After washing, 20 ng human C4 was added to each well and incubated at 37°C for 90 min. After washing, wells were sequentially incubated with rabbit anti-human C4c antibody and biotinylated anti-rabbit antibody followed by alkaline phosphatase conjugated anti-rabbit antibody (ABP-AP, Vector laboratories). Samples were developed using *p-*NPP substrate and absorbance was measured at 405 nm. Assays were performed in triplicate using 4 samples each from PBS control or WTA immunized MBL KO mice. WT mice were not included as the purpose of these experiments was to examine the complement activation activity of anti-WTA IgG, which competes with MBL for binding and complement activation [21].

### *Ex vivo* whole blood assays

Whole blood assays were performed using previously described methods with minor modifications [12]. Bacteria, MW2 CA-MRSA and COL HA-MRSA strains, were cultured and used as described above. Naïve MBL KO and WT mice were euthanized and blood was collected by cardiac puncture with hirudin (Sigma-Aldrich) as an anticoagulant. 25 µl of whole blood was mixed with 20 µl of mouse serum obtained from MBL KO mice immunized with WTA or PBS control. Four serum samples from each WTA or PBS control immunized mouse were used. These mixtures were added to 5 µl of bacterial suspension containing 2500 cfu and incubated for 2 hrs at 37° C. Samples were serially diluted in EDTA-saline solution and then cultured on TSA plates for colony counting as described above. Assays were performed in duplicate and repeated twice.

### Statistical analysis

All data were analyzed using JMP software (SAS Institute, Inc). The methods of statistical analysis were indicated in each figure. p values less than 0.05 were considered to be significant.

## Results

### MBL deficiency is relatively protective against MW2 CA-MRSA infection although there is little effect on COL HA-MRSA infection in mice

We have previously reported that MBL KO mice are susceptible to infection with certain pathogens, including antibiotic sensitive strains of *S. aureus* [12]. In order to determine the effect of MBL on susceptibility to infection with MW2 CA-MRSA and COL HA-MRSA, MBL KO and WT mice were evaluated using the abscess formation model as described in the Materials and Methods (Figure S1). Of the organs examined – kidney, liver, lung, heart, and spleen – abscesses were predominantly observed in the kidney with both MW2 CA-MRSA and COL HA-MRSA infection. The incidence of kidney abscess was similar in both MBL KO and WT mice in COL HA-MRSA infection (Table 1). In contrast, following MW2 CA-MRSA infection, no abscess formation was observed in MBL KO mice although 42% of WT mice had kidney abscess (Table 1). These results demonstrate that the kidney is susceptible to abscess formation, and that MBL has a variable role depending on the *S. aureus* strain, in that MBL deficiency has little effect on COL HA-MRSA infection whereas it contributes to decreased MW2 CA-MRSA infection [10].

**Table 1:** Abscess formation in systemic MRSA infection.

Bacterial strains	COL HA-MRSA		MW2 CA-MRSA	
Mouse strains	WT	MBL	WT	MBL
Kidneys/Total (%)	8/18 (44)	10/18 (56)	10/24 (42)	0/24 (0)*
Mice/Total (%)	5/9 (55)	7/9 (78)	8/12 (66)	0/12 (0)*

* indicates p<0.0001 (Likelihood ratio) compared to WT in MW2 CA-MRSA infection.

### Intradermal immunization with WTA elicits and augments anti-WTA IgG responses in both MBL sufficient and MBL deficient mice

We have previously found that healthy people have detectable titers of WTA specific IgG in serum [21]. Moreover, we have also demonstrated that purified anti-WTA IgG competes with MBL for recognition and phagocytosis of *S. aureus* [21]. It has been reported that subcutaneous injection of a mixture of WTA and an irritant induces abscess formation in murine skin, suggesting that WTA plays a role in abscess formation [10]. Based on these observations, we hypothesized that WTA immunization would induce an anti-WTA IgG response, resulting in reduced susceptibility to MRSA infection and abscess, regardless of MBL deficiency in mice. Therefore, we next examined anti-WTA IgG response following intradermal WTA immunization in both MBL KO and WT mice. The intradermal injection route was chosen based on recent reports of induction of protective immunity against several pathogens using this route of immunization [16], [17], [18], [19].

**Figure 1:**
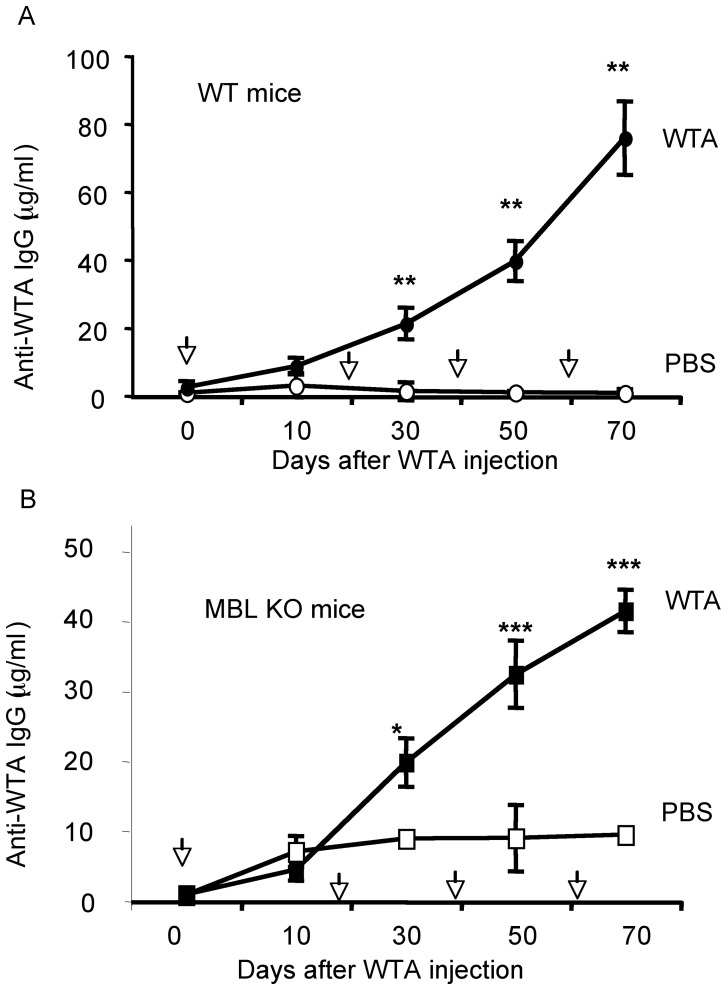
WTA immunization elicits and augments WTA-specific IgG in WT (1A) and MBL KO mice (1B). PBS control or 5 µg of WTA was intradermally injected on days indicated by black arrows. No adjuvant was used throughout the study. Four WT mice per group and 5 MBL KO mice per group were used. Anti-WTA IgG serum titers are plotted as mean **±** SD. *, ** and *** indicates p<0.01, 0.001 and 0.0005 (Welch's test).

Prior to immunization (day 0), naïve WT and MBL KO mice had undetectable or very little anti-WTA IgG titers (Figures 1A and 1B, respectively). In WT mice, anti-WTA IgG titers significantly increased after the second WTA immunization and continued to increase until after the fourth immunization (Figure 1A). An anti-WTA IgG response was not observed following injection of PBS control (Figure 1A). A similar increase in anti-WTA IgG titer was observed in MBL KO mice after the second immunization with WTA but not with PBS control (Figure 1B). Anti-WTA IgG titers were incrementally increased in both WT and MBL KO mice with serial immunizations. (Figure 1A and 1B, respectively). No sign of rash or abscess formation was observed at the injection sites throughout the study, suggesting multiple WTA injections did not induce any local adverse reaction. In addition, all mice gained weight by 3–8% and appeared to be healthy and without signs of illness or unusual behavior during and after the immunizations, suggesting that WTA immunization did not have an adverse effect, physically or mentally, in mice. These results demonstrate that naïve mice, raised in a pathogen free environment, do not have anti-WTA IgG in serum and that intradermal WTA immunization both elicited and augmented an anti-WTA IgG response, independent of the presence of MBL.

### Intradermal WTA immunization reduces susceptibility to MRSA infection independent of MBL

We tested whether the enhanced anti-WTA IgG response from intradermal WTA immunization confers protection from MRSA infection. We used the abscess formation model following systemic infection of sublethal inoculums of two MRSA strains, MW2 CA-MRSA and COL HA-MRSA, as described above.

**Figure 2:**
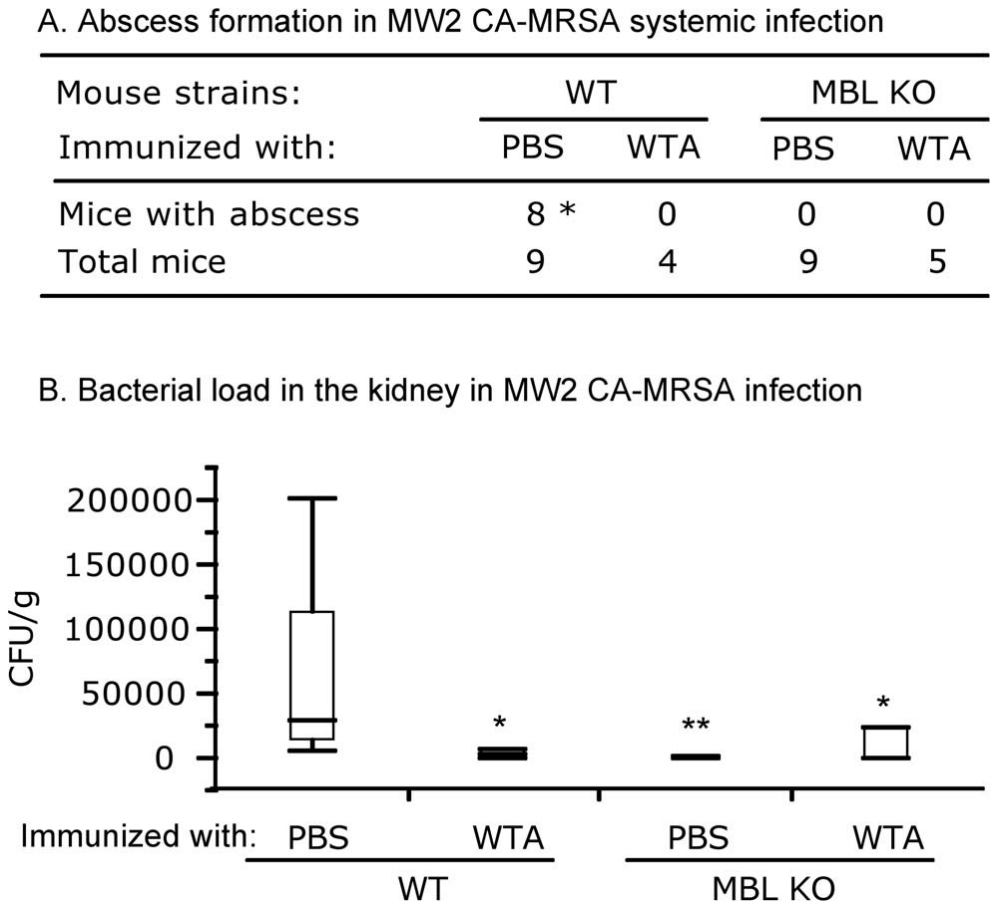
WTA immunization reduces MW2 CA-MRSA infection in WT mice while no difference is seen in the absence of MBL. (2A) Abscess formation. Mice immunized with PBS control or WTA were infected 20 days after the last immunization. Abscess formation was examined on day 10 following systemic infection with MW2 CA-MRSA as detailed in the Materials and Methods. Abscess formation is expressed as numbers of mice with abscess and total mice in each group. * indicates p<0.0001 against all other groups (Likelihood Ratio). (2B) Bacterial load in the kidney. Bacterial titers were measured in homogenates of two combined kidneys and are expressed as cfu/g of kidneys in a box plot. Numbers of mice used are as in figure 2A. * and ** indicates p<0.05 and p<0.001, respectively compared to WT immunized with PBS control (Nonparametric comparisons for each pair by Wilcoxon methods).

We first tested MW2 CA-MRSA infection. Abscess formation was predominantly observed in the kidney of PBS control immunized WT mice whereas no abscess formation was observed in WTA-immunized WT mice (Figure 2A). In MBL KO mice, intradermal WTA immunization had little effect in abscess formation because no abscess formation was observed in both PBS control immunized MBL KO mice (Figure 2A). Although abscess formation was not observed in WTA-immunized WT and MBL KO mice using MW2 CA-MRSA, culture of kidney homogenates detected bacteria, suggesting that there was still infection. Nevertheless, bacterial loads were significantly reduced in WTA immunized WT mice while they were similar between PBS control and WTA immunized MBL KO mice (Figure 2B).

**Figure 3:**
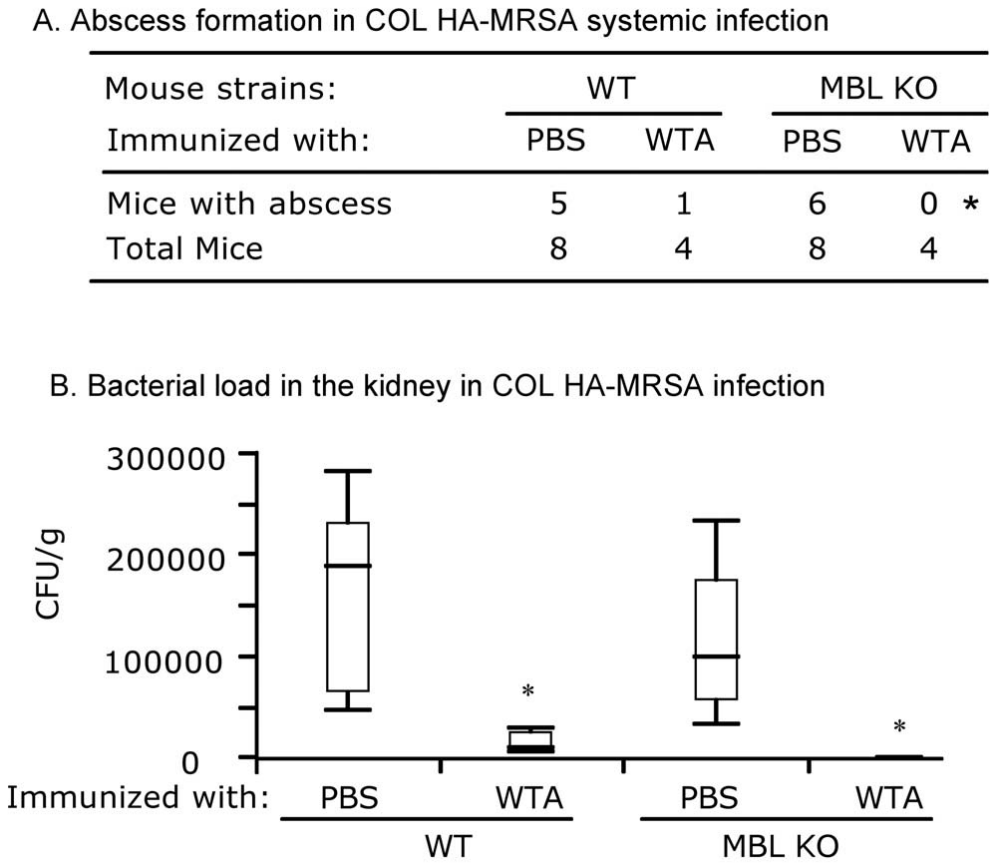
WTA immunization reduces COL HA-MRSA infection in both MBL sufficient and MBL deficient mice. (3A) Abscess formation. Mice immunized with PBS control or WTA were infected at 20 days after the last immunization. Abscess formation was examined on day 10 following systemic infection with COL HA-MRSA as detailed in the materials and methods. Abscess formation is expressed as numbers of mice with abscess and total mice in each group. * indicates p<0.001 against WTA immunized groups (Likelihood ratio test). (3B) Bacterial load in the kidneys. Bacterial titers were measured in homogenates of two combined kidneys and are expressed as cfu/g of kidneys in a box plot. Numbers of mice used are as in figure 3A. * indicates p<0.01 compared to WT immunized with PBS control (Nonparametric comparisons for each pairs by Wilcoxon methods).

Next, we tested COL HA-MRSA infection. Intradermal WTA immunization reduced abscess formation in both MBL KO and WT mice although statistical significance was obtained only for the MBL KO mice group (Figure 3A). The bacterial loads of COL HA-MRSA were significantly reduced in both MBL sufficient and MBL deficient mice that were immunized with WTA (Figure 3B). These results demonstrate that intradermal WTA immunization is effective in reducing infection, as assessed by measurement of abscess formation and bacterial loads, with both MW2 CA-MRSA and COL HA-MRSA, independent of the presence of MBL.

### Anti-WTA IgG containing serum activates complement and inhibits MRSA growth in *ex vivo* whole blood assays

The complement activation activity of anti-WTA IgG in serum was assayed by measuring C4c deposition, as described above. Since C4C deposition is mediated by both the classical and the lectin pathways, the lectin pathway was minimized by using anti-WTA IgG containing serum from MBL KO mice, in which the lectin pathway activity is sharply curtailed due to the absence of MBL. C4c deposition activity (mean OD reading ± SD) was 0. 499±0.056 and 0.388±0.037 (p<0.002 by Student's t test) for WTA and PBS control immunized serum samples, respectively. These results demonstrate that anti-WTA IgG containing serum is able to initiate the classical complement pathway on the presence of WTA.

Whole blood contains all of the soluble factors and a range of effector cells for complement activation. Complement activation and consequent inhibition of bacterial growth is a meaningful assay of complement activity, including bactericidal activity, as we have previously reported [12], [21]. Therefore, we tested the ability of anti-WTA serum to limit MRSA growth in *ex vivo* whole blood assays. To analyze the effects of MBL in this setting, we examined bacterial growth in a mixture of anti-WTA IgG containing serum obtained from WTA-immunized MBL KO mice and whole blood of naïve WT and MBL KO mice.

**Figure 4:**
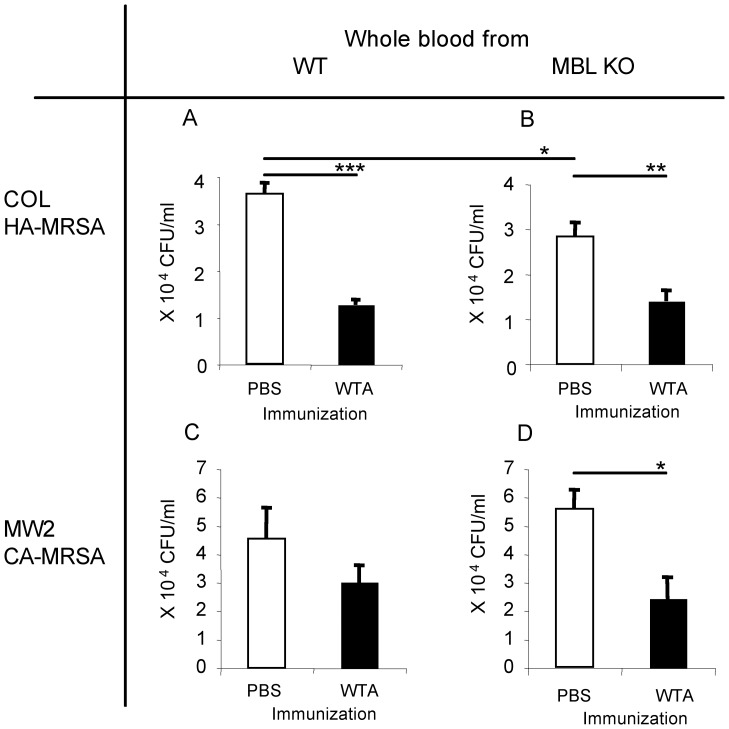
Serum from WTA-immunized mice inhibits bacterial growth in whole blood assays *ex vivo*. Two MRSA strains, COL HA-MRSA (4A and 4B) and MW2 CA-MRSA, (4C and 4D) were used. Bacteria were incubated with whole blood from MBL KO (4A and 4C) or WT mice (4B and 4D) with serum from MBL KO mice immunized with either PBS control or WTA, as described in the Materials and Methods. Bacterial titers are expressed as mean **±** SD in each group of four samples. Each sample was measured in duplicate and the average measurement was used for statistical analysis. Representative results from two repeated experiments are shown. *, **, and *** indicate p<0.05, 0.001, and 0.0001 (Student's t-test), respectively.

Compared with PBS control immunized serum, anti-WTA IgG containing serum significantly reduced bacterial growth of COL HA-MRSA in whole blood of both WT and MBL KO mice (Figure 4A and 4B, respectively). Similar bacterial growth inhibition was obtained using MW2 CA-MRSA, with statistical significance for MBL KO whole blood (Figure 4C and 4D). Comparison of PBS control immunized serum from WT and MBL KO mice showed significant reduction in COL HA-MRSA growth by MBL KO serum while no significant difference was observed for MW2 CA-MRSA (Figure 4). These results show that anti-WTA IgG containing serum is able to inhibit MRSA growth and reduce the numbers of MRSA in whole blood independent of the presence of MBL.

## Discussion

Our recent finding that healthy people have circulating anti-WTA IgG led us to hypothesize that WTA immunization would result in an anti-WTA response. We now report that this hypothesis is correct, with the results provided here demonstrating that intradermal WTA immunization elicits and augments anti-WTA IgG production in mice, without use of an adjuvant. These results provide *in vivo* evidence that WTA itself is immunogenic. The incremental increase in IgG titer with serial WTA immunization suggests that WTA mediates a T cell-dependent antibody response, which requires B cell germinal center formation [30]. Our unpublished observations show that intradermal WTA immunization results in germinal centers (B220 and PNA double positive splenocytes) adjacent to T cell zones in spleen. Taken together, these observations suggest that WTA is a T cell-dependent antigen, even though polysaccharide antigens have previously been regarded as T cell-independent [31].

We note that we used highly purified WTA that does not contain lipoproteins or LTA. The former was not present due to an *lgt* gene mutation and the latter was efficiently removed during our purification process [21], [22]. Although we do not know whether these molecules modulate anti-WTA antibody responses, they do induce inflammation, whereas WTA does not [10], [32], [33]. Further investigation of the inflammatory response to WTA may help to better understand the mechanisms of anti-WTA IgG induction.

It has been reported that an abscess develops at the site of subcutaneous injection when WTA and dextran are administered together [10]. In contrast, we used an intradermal injection without any adjuvant or other foreign material and did not observe abscess formation even after multiple WTA injections. It is well established that different host responses can be elicited from the same antigen by manipulation of multiple factors, including adjuvant or irritants such as dextran, and different routes of injection, such as intradermal or subcutaneous [16], [17], [18], [19]. Recent studies have demonstrated that intradermal administration can produce a more effective immune response compared to other routes of administration [34], [35], [36]. For example, intradermal immunization resulted in protective immunity with tuberculosis and hepatitis C virus vaccines, while subcutaneous injection failed to elicit a response or was less effective [17].

In the results presented here we find that the anti-WTA IgG response is not affected by the absence of MBL, the deficiency of which is a common primary immunodeficiency in humans. In contrast, in our prior study, MBL deficiency enhanced IgG responses to certain antigens [27]. Of major significance for purposes of clinical translation, the enhanced anti-WTA IgG response confers protection from infection with both COL HA- and MW2 CA-MRSA, regardless of MBL status. As both of these strains were isolated from humans, we hypothesize that anti-WTA IgG may contribute to protection from *S. aureus* infection in humans. The results presented here demonstrate that intradermal immunization with highly purified WTA effectively induces adaptive immunity in mice, and we hypothesize that this will be obtain in humans also.

An unexpected new finding in the current study is that MBL deficiency relatively protects mice from infection with MW2 CA-MRSA, as shown by decreased kidney abscess formation, while it seems to have little effect on susceptibility to COL HA-MRSA infection. MW2 is widely used as a strain of CA-MRSA (also known as USA400), although *S. aureus* USA300 is now the most commonly isolated CA-MRSA strain in the US. It is not known whether MBL deficiency is protective from other strains of MRSA bacteria. Nevertheless, these findings raise the possibility that some strains of MRSA could utilize MBL, an opsonin, to evade the host defense system. This possibility has been proposed as a mechanism for some intracellular pathogens, such as tuberculosis and leishmaniasis, although the precise molecular mechanisms are not fully understood and further investigations are required [37], [38], [39]. These findings may explain why MBL deficiency has not been strongly associated with *S. aureus* infection in humans, particularly in adults, and the exact role of MBL in relation to MRSA associated virulence factors requires further investigation [12], [13], [14].

We have chosen a model of abscess formation following systemic inoculation to assess infection because abscess formation requires bacterial tissue adhesion and invasion, which WTA has been shown to promote [7], [9], [40], [41]. Also, increased expression of WTA and resultant thickening of the cell wall may contribute to antibiotic resistance, in addition to an array of other virulence factors, such as penicillin binding proteins and efflux pumps [42], [43], [44], [45], [46]. In any case, the results from our investigations suggest that targeting WTA is a promising approach to controlling *S. aureus* infection.

The results presented here also suggest a mechanism of the microbicidal action of anti-WTA IgG that limits bacterial growth in whole blood. WTA has been implicated in facilitating bacterial attachment to host cells and tissues. Data presented here, from whole blood assays, suggest that anti-WTA IgG eliminates bacteria in blood prior to the bacteria attaching to cells and invading tissues. Our previous studies have also shown that human anti-WTA IgG increases opsonophagocytosis, which is correlated with increased bacterial killing [21], [22]. These studies also have shown that anti-WTA IgG, upon binding to *S. aureus*, activates the classical complement pathway, and so anti-WTA IgG contributes directly to bacterial killing by initiating membrane attack complex formation. This current study also provides evidence that anti-WTA IgG containing serum is able to activate the classical pathway. Consequently, our data along with previously reported results, suggest that anti-WTA IgG is involved with direct bactericidal action and opsonophagocytic killing.

In summary, we present *in vivo* evidence that highly purified WTA (with undetectable lipoproteins and peptidoglycans) is immunogenic in a T cell-dependent manner, resulting in an anti-WTA IgG response that is effective against MRSA infection. The efficacy of anti-WTA immunity is not affected by MBL sufficiency, although the role of MBL in MRSA infection is variable, in that MBL deficiency is protective against MW2 CA-MRSA infection while MBL inhibits COL HA-MRSA in whole blood. These observations lead to the hypotheses that naturally occurring anti-WTA adaptive immunity reduces susceptibility to infection to *S. aureus*, particularly in the adult population, and that high serum levels of MBL in humans may be a risk factor for infection with some strains of *S. aureus*. However, as *S. aureus* exposure in humans is multifactorial, involving skin and mucosa, further investigation is warranted to understand the precise mechanisms by which anti-WTA immunity is naturally mounted. It is also possible that sub-components of WTA may modulate its immunogenicity, and so studies of WTA variants may reveal more precisely the mechanisms of anti-WTA immunity.

Lastly, it is also possible that a similar immunization protocol, using purified WTA with an appropriate (or possibly no) adjuvant, may maximize anti-WTA IgG response and provide protection from *S. aureus* infection in humans. It may also be possible that anti-WTA IgG could serve as a passive immunization therapy to treat patients with established and difficult to treat *S. aureus* infections.

## Supporting Information

Figure S1 Supplemental figure 1. Kidneys with or without abscess are shown.
(PPT)
