# Supplementary material for: Intradermal Immunization with Wall Teichoic Acid (WTA) Elicits and Augments an Anti-WTA IgG Response that Protects Mice from Methicillin-Resistant Staphylococcus aureus Infection Independent of Mannose-Binding Lectin Status
Source: PLoS One. 2013 Aug 2;8(8):e69739. doi: 10.1371/journal.pone.0069739 (PMC3732247; doi:10.1371/journal.pone.0069739)
Supplement: Figure S1 — Supplemental figure 1. Kidneys with or without abscess are shown. (PPT) [file pone.0069739.s001.ppt]

## Slide 1
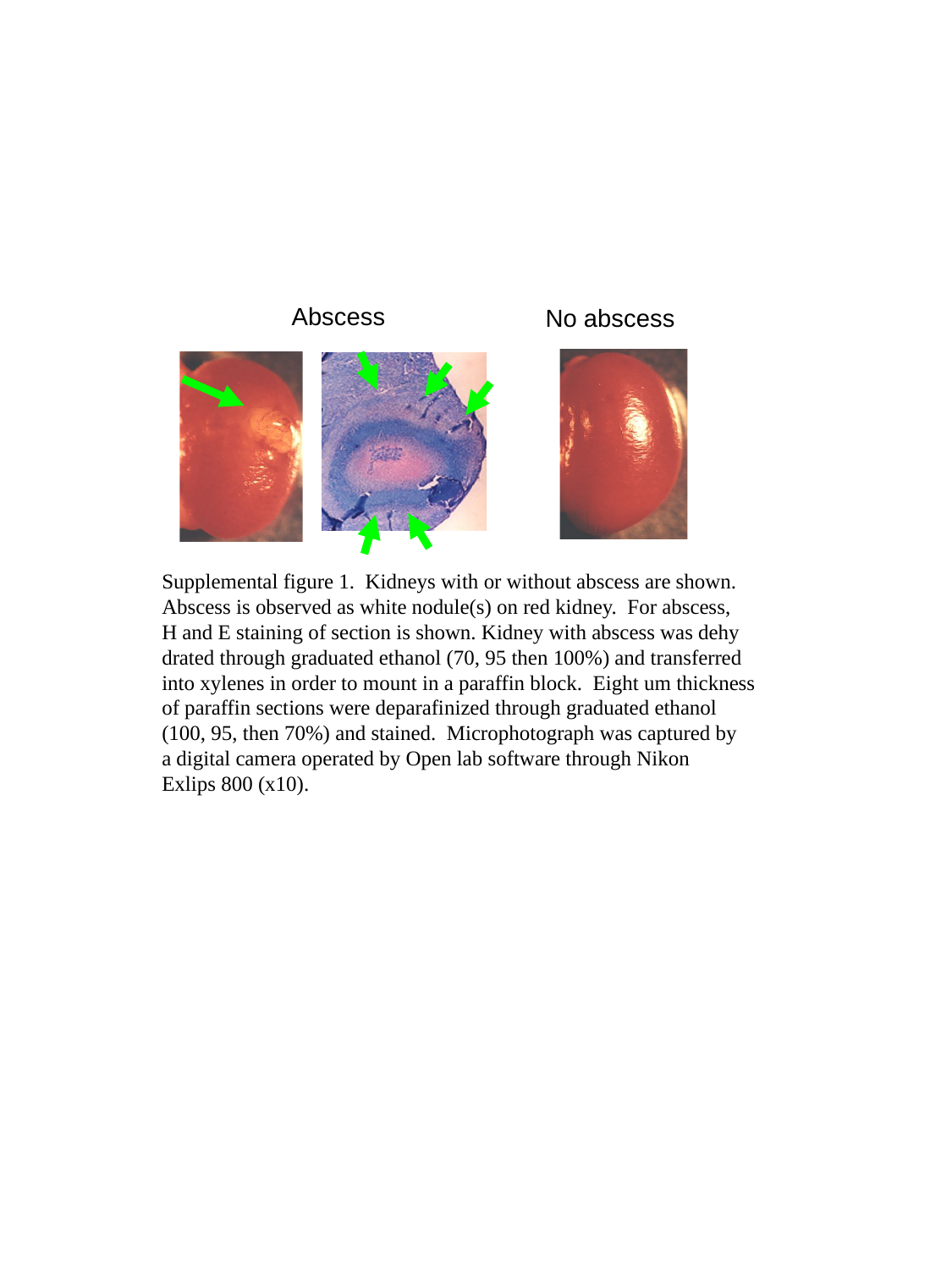

Abscess
No abscess
Supplemental figure 1. Kidneys with or without abscess are shown.
Abscess is observed as white nodule(s) on red kidney. For abscess,
H and E staining of section is shown. Kidney with abscess was dehy
drated through graduated ethanol (70, 95 then 100%) and transferred
into xylenes in order to mount in a paraffin block. Eight um thickness
of paraffin sections were deparafinized through graduated ethanol
(100, 95, then 70%) and stained. Microphotograph was captured by
a digital camera operated by Open lab software through Nikon
Exlips 800 (x10).
